# Laser-Fluence Effect on Morphological Evolution and Optical Response of Ge Microcones Grown on Si by Pulsed Laser Deposition

**DOI:** 10.1021/acsomega.6c07433

**Published:** 2026-08-25

**Authors:** Francisco Javier Altamirano-García, Rosalba Castañeda-Guzmán, Mónica Estefanía Martínez-Saucedo, Erick Benítez-Flores

**Affiliations:** † Instituto de Ciencias Aplicadas y Tecnología, UNAM, A. P. 70-186, Ciudad México 04510, México; ‡ Facultad de Ciencias, UNAM, A.P.70-399, Ciudad de México 04510, México

## Abstract

Microstructured germanium (Ge) coatings on silicon (Si) are promising for enhancing light–matter interactions in optoelectronic devices. This work reports a single-step pulsed laser deposition (PLD) route for fabricating crystalline Ge conical microstructures and nanostructures on Si. A review of the available literature indicates that this microconical morphology has not been previously reported in Ge/Si systems grown by PLD. Laser fluence is the key parameter governing the morphology of the Ge microstructures. Depositions at 1, 2, and 3 J/cm^2^ produce distinct populations of conical structures, ranging from nanocones (190 ± 10 nm in height and 360 ± 10 nm in base diameter) at 1 J/cm^2^, to intermediate microcones (1.4 ± 0.1 μm in height and 2.8 ± 0.1 μm in base diameter) at 2 J/cm^2^, and well-developed microcones (5.8 ± 0.1 μm in height and 16.5 ± 0.1 μm in base diameter) with higher surface density at 3 J/cm^2^. UV–vis measurements reveal a morphology-dependent optical response, with broadband reflectance reduced to 30–40% over the 550–1200 nm wavelength range. These results show that laser fluence is an easy and effective way to control both the shape and optical properties of Ge microcones, making single-step PLD a scalable method for creating light-trapping Ge/Si interfaces in photonic and optoelectronic devices.

## Graphical abstract

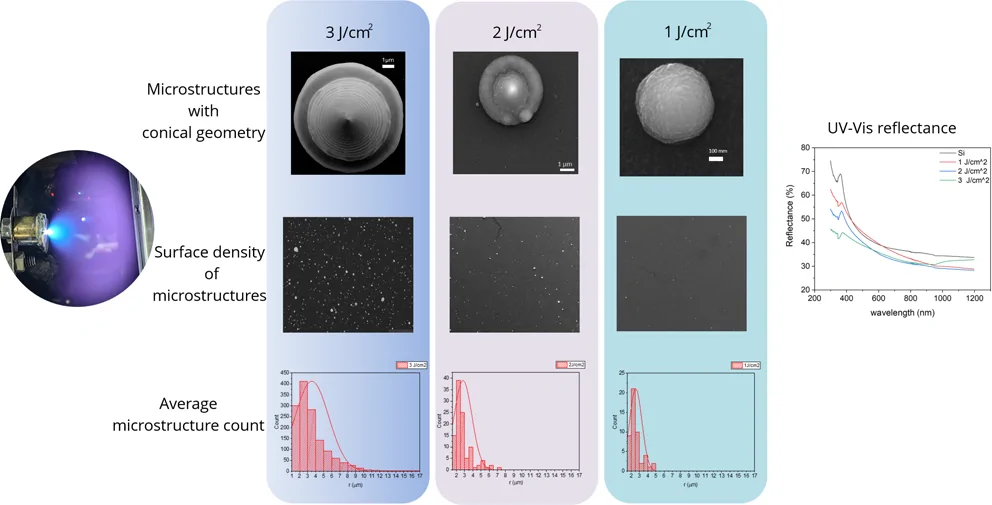

## 1 Introduction

Silicon (Si) and germanium (Ge) belong to group IV of the periodic table and therefore share several structural and electronic characteristics. Both semiconductors are widely used in the electronics industry due to their band gap energy: for Si, it is 1.12 eV (indirect), whereas for Ge, it is 0.66 eV (also indirect), in bulk at room temperature. These properties make them attractive for the fabrication of optoelectronic components such as medical sensors, light-emitting devices, and photovoltaic cells.^1,2^ In many of these applications, the goal is for the silicon to absorb the maximum amount of light, which has been achieved by coating the surface with thin Ge films or with Ge microstructures on Si^3^ since the efficiency of these devices depends on the properties of their surface.^4,5^ Furthermore, Ge has attracted renewed interest for applications in photonics and solar energy, particularly due to its potential to improve photovoltaic efficiency through spectral conversion processes, such as red-shift or down-shift, in which ultraviolet photons are converted into visible or infrared photons.^6^

Deposition of Ge on Si can be achieved using various techniques, including vapor–liquid–solid (VLS), molecular beam epitaxy (MBE), chemical vapor deposition (CVD), and magnetron sputtering.^7–9^ Among these, pulsed laser deposition (PLD) offers unique advantages: it enables high-purity films with controlled stoichiometry,^10–12^ operates at relatively low substrate temperatures, and allows single-step fabrication of complex micro/nanostructures without catalysts^13^ or multiple processing stages.^14^ However, PLD is often associated with the formation of ejected particulates (splashing), which can degrade film quality. While many studies have focused on suppressing this effect,^15,16^ we instead exploit it to generate well-defined three-dimensional Ge microcones directly on Si, turning a common drawback into a morphological control tool.

When Ge is deposited on Si, the 4.2% lattice mismatch^17,18^ typically drives the formation of pyramidal or dome-shaped islands. Previous studies have reported square-based pyramids with dimensions ranging from a few monolayers to several nanometers,^19^ which may evolve into dome-shaped structures during growth.^19–22^ In contrast, this work reports the growth of microcones centered on circular bases, with dimensions ranging from a few hundred nanometers to several micrometers. This morphology differs significantly from previously documented Ge/Si island structures, suggesting a distinct growth mechanism.

Based on these considerations, it is hypothesized that laser fluence (J/cm^2^) is the primary and tunable parameter governing the size and surface density (cm^–2^) of Ge microcones grown on Si by pulsed laser deposition (PLD), and that these morphological changes systematically modulate the optical reflectance of the Ge/Si interface. This hypothesis was evaluated by depositing Ge films on Si (001) substrates at three laser fluences (1, 2, and 3 J/cm^2^), with all other deposition parameters held constant. The resulting microstructures were characterized by scanning electron microscopy (SEM), X-ray diffraction (XRD), and UV–vis reflectance spectroscopy to establish quantitative relationships among laser fluence, morphology, crystallinity, and optical response.

This study demonstrates a single-step, lithography-free and chemical-etching-free PLD route for the direct fabrication of crystalline Ge microcones with a distinctive circular-base morphology. To the best of our knowledge, Ge microcones with this morphology have not previously been reported using PLD. The study further establishes a quantitative correlation between laser fluence and the resulting microcone dimensions, surface density, and broadband optical response. Unlike previous studies that primarily address Ge island evolution or seek to suppress splashing during PLD, the present work intentionally exploits this phenomenon as a morphology-control mechanism, providing a simple, scalable strategy for engineering light-trapping Ge/Si interfaces for optoelectronic and photovoltaic applications.

## 2 Materials and Methods

Si (001) substrates were cleaned with water and 10% Alconox, then rinsed with deionized water, immersed in distilled water, and ultrasonically treated for 5 min. The procedure was repeated with acetone and isopropanol, after which the substrates were dried with an air blower. Targets were mounted inside the vacuum chamber, and substrates were fixed on a holder and positioned facing the target. The target-to-substrate distance was set to 4.00 ± 0.01 cm.

An Ekspla Nd:YAG laser model NL300 operating at 1064 nm, using the second harmonic (λ = 532 nm) at 10 Hz with a 7 ns pulse width, was used to irradiate the Good Fellow 99.99% Ge target. The beam was focused to a 0.004 cm^2^ spot, and pulse energies of 4, 8, and 12 mJ were used, corresponding to fluences of 1, 2, and 3 J/cm^2^, respectively. The chamber was evacuated to a base pressure of 1.03 × 10^–3^ Pa. During deposition, substrates were heated to 300 °C^23^ (Figure 1). Ge films were deposited using 12000 pulses (20 min).

**Figure 1:**
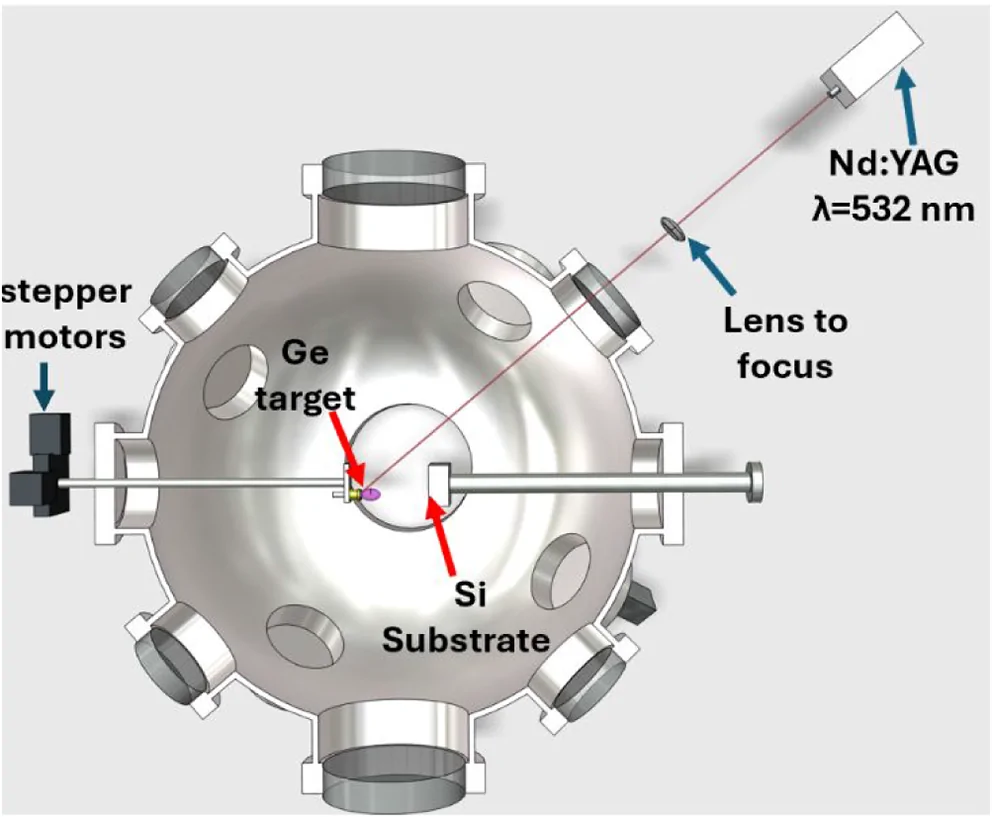
Schematic diagram of the vacuum chamber for the PLD process.

Samples were analyzed using a JEOL JSM-7800F Scanning Electron Microscope. ImageJ was used to determine surface density (ρ) of microstructures and to characterize their dimensions. XRD analysis was performed using a Rigaku Ultima IV diffractometer with Cu Kα radiation at a scan speed of 1°/min to characterize the crystallography of the samples. Reflectance was measured using a Cary-5000 UV–vis spectrometer.

The possible formation mechanism of the observed microcones is discussed in the Results and Discussion section, based on the morphological features revealed by SEM.

## 3 Results and Discussion

A hybrid growth mechanism is proposed to explain the formation of the Ge microcones, involving two concurrent processes inherent to pulsed laser deposition (PLD): (i) ballistic deposition of atomic and ionic species from the ablation plume, and (ii) redeposition of molten Ge droplets (splashing) ejected from the target.^13^ Cross-sectional SEM images reveal well-defined terraced morphologies on the larger cones (Figure 2a), characteristic of layer-by-layer or step-flow growth driven by adatom diffusion on the heated substrate. In contrast, pancake-like splashing features observed adjacent to the cones indicate that molten Ge droplets also reach the substrate surface and likely serve as preferential nucleation sites.^24^

**Figure 2:**
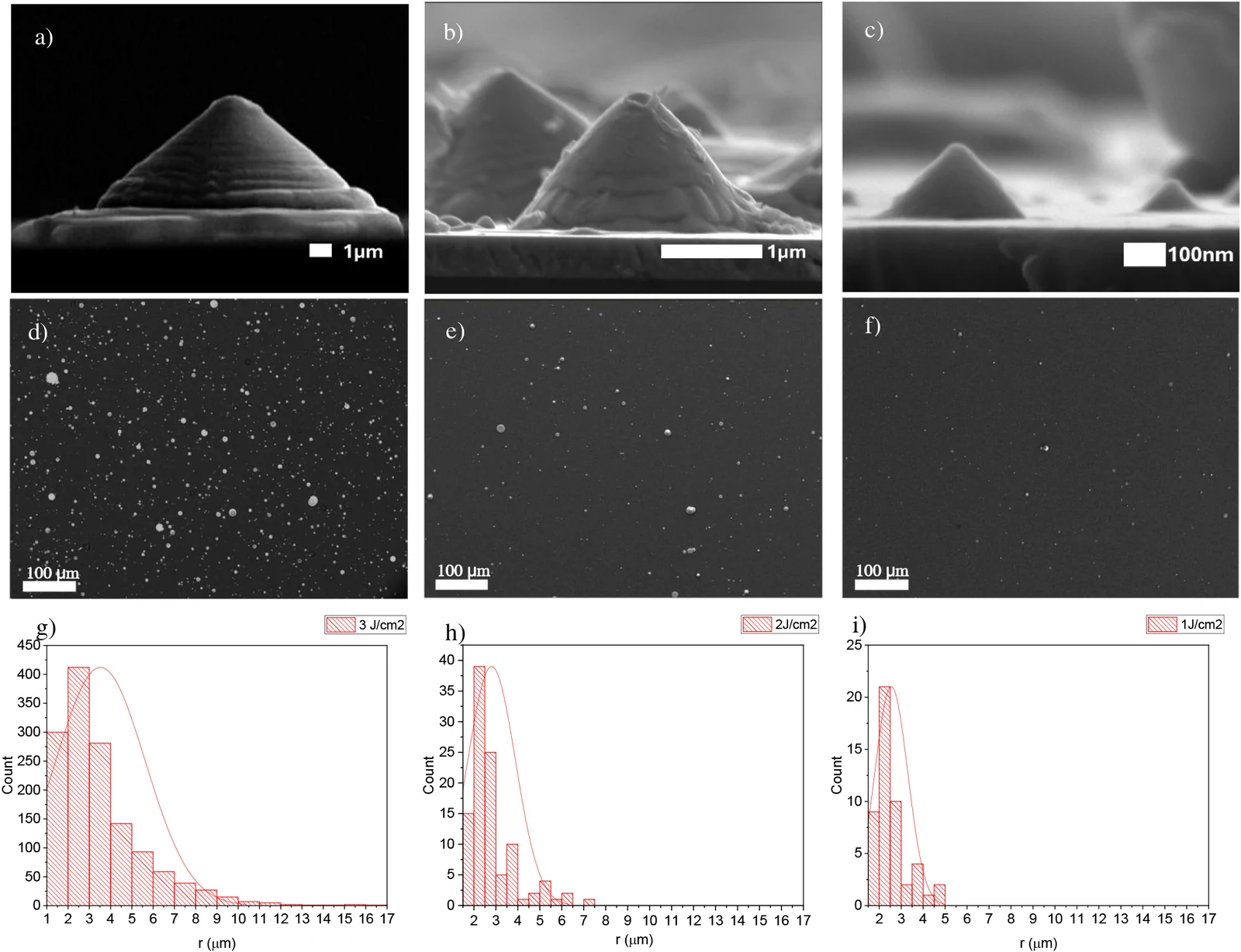
Representative SEM images of Ge microcones deposited on Si at different laser fluences are shown. Cross-sectional views reveal a progressive reduction in cone dimensions with decreasing fluence: (a) 3 J/cm^2^ base diameter of 16.5 ± 0.1 μm; (b) 2 J/cm^2^ base diameter of 2.8 ± 0.1 μm; and (c) 1 J/cm^2^ base diameter of 360 ± 10 nm. Surface-view images (d–f) show a similar decrease in surface density of the microstructures as fluence decreases, from a high-density distribution with visible splashing crowns at 3 J/cm^2^ to a sparse population of mostly sub-10 μm features at 1 J/cm^2^. Images (g–i) are Size-distribution histograms of the microcone radius (r) for Ge structures grown at laser fluences of (g) 3 J/cm^2^, (h) 2 J/cm^2^, and (i) 1 J/cm^2^. The solid red curves represent the fitted probability density functions, showing a progressive shift toward smaller radii as fluence decreases.

Based on these observations, the microcones are proposed to form through the impact and rapid solidification of molten Ge droplets, which then serve as seeds for the accumulation of vapor-phase species.^24^ The continuous arrival of adatoms promotes preferential vertical growth, yielding the terraced morphology observed in the SEM images.^13^ This sequential mechanism—droplet-mediated nucleation followed by adatom-driven overgrowth—is consistent with the systematic increase in microcone size and surface density with laser fluence, as higher fluence increases both the droplet population and the adatom flux.^13,25^ Conversely, at lower fluences (1 J/cm^2^), reduced droplet density and lower supersaturation restrict growth to nanoscale dimensions^25^ show in Figure 2a–c. Although the initial nucleation stage cannot be directly resolved in the present experiments, the proposed mechanism is supported by the coexistence of splashing features and terraced structures and is consistent with previous reports describing PLD-grown microcolumns and cone-like morphologies in related material systems.^13,24,25^

Figure 2a–f presents SEM images of Ge microcones grown at different laser fluences, showing that both the size and surface density of the structures increase with fluence. Cross-sectional images reveal a progressive reduction in dimensions from 5.8 ± 0.1 μm height and 16.5 ± 0.1 μm base diameter at 3 J/cm^2^ to 1.4 ± 0.1 μm and 2.8 ± 0.1 μm at 2 J/cm^2^, and finally to nanoscale features of 190 ± 10 nm height and 360 ± 10 nm base diameter at 1 J/cm^2^. This trend indicates that higher fluence enhances material ablation, species kinetic energy, and mass transport, promoting nucleation and vertical growth of larger three-dimensional cones, whereas lower fluence limits growth. The terraced morphology observed in larger cones suggests sequential deposition. Statistical analysis of cone size evolution was performed on 15 independent SEM images (area of (5 ± 0.2) × 10^–3^ cm^2^ each) per fluence, with cone radii measured using ImageJ.

The Ge microcones obtained in the present study can be compared with Ge microstructures fabricated by alternative approaches reported in the literature. For example, Zhong and Bauer^14^ employed holographic lithography with double exposure, followed by reactive ion etching (RIE), to fabricate site-controlled, size-homogeneous Ge islands on prepatterned Si(001) substrates. Although this approach provides excellent positional control and high morphological uniformity, it relies on multiple processing steps, including lithographic patterning and subsequent etching. In contrast, surface texturing methods based on laser irradiation or plasma etching have been reported to generate Ge surface protrusions and roughened microstructures through localized material removal rather than deposition. For instance, Zheleznov et al.^26^ observed laser-induced surface microstructures on Ge single crystals following nanosecond UV irradiation, whereas Jiang et al.^27^ demonstrated that kinetic instabilities during plasma etching lead to the formation of irregular surface protrusions with limited control over their spatial distribution and morphology. Unlike these approaches, the present PLD method directly exploits the intrinsic dynamics of laser ablation and droplet-mediated growth to fabricate self-organized Ge microcones on Si in a single deposition step, eliminating the need for prepatterning, masks, wet chemical processing, or postgrowth surface modification. This difference highlights the simplicity, scalability, and versatility of the proposed approach for producing well-defined Ge microcone arrays for light-management applications.

Figure 2g–i presents histograms of the microcone radius distributions for each deposition condition, allowing identification of representative trends for each condition.

At a fluence of 1 J/cm^2^, the distribution of radii is concentrated below 5 μm, with a mean radius of 2.4 ± 0.75 μm and an average surface density of (1.07 ± 0.14) × 10^4^ cm^–2^. This suggests a regime near the ablation threshold.

Increasing fluence to 2 J/cm^2^ slightly broadens the distribution; the average radius is 2.8 ± 1 μm, and the average surface density is (2.09 ± 0.32) × 10^4^ cm^–2^, indicating a greater amount of ejected material. Finally, at 3 J/cm^2^, the distribution shows an extended tail, indicating the formation of larger microcones; in this case, the average radius is 3.88 ± 0.77 μm, and the surface density is (5.31 ± 0.29) × 10^4^ cm^–2^. Therefore, the density increases significantly.

Microscopy also reveals pancake-type splashing caused by high-temperature Ge droplets impacting the substrate. Laser fluence was identified as the key parameter controlling the formation of Ge microcones deposited by pulsed laser deposition. Increasing fluence systematically increases both the surface density and the characteristic dimensions of microcones, reflecting a transition from near-threshold ablation to regimes dominated by molten-material ejection and redeposition. Thus, laser fluence enables partial control over the average size and areal density of the structures.

Figure 3, Ge-coated Si samples do not exhibit the interference fringes typically observed in uniform planar films.^28^ Instead, microcone morphology suppresses coherent reflections through surface roughness, diffuse scattering, and optical-path inhomogeneity. The absence of Fabry–Pérot oscillations indicate that the deposited Ge layers behave optically as textured graded interfaces rather than homogeneous thin films.^29,30^ Increasing laser fluence further reduces reflectance,^31,32^ indicating that larger and denser microcones enhance broadband light trapping through combined scattering and refractive-index grading.

**Figure 3:**
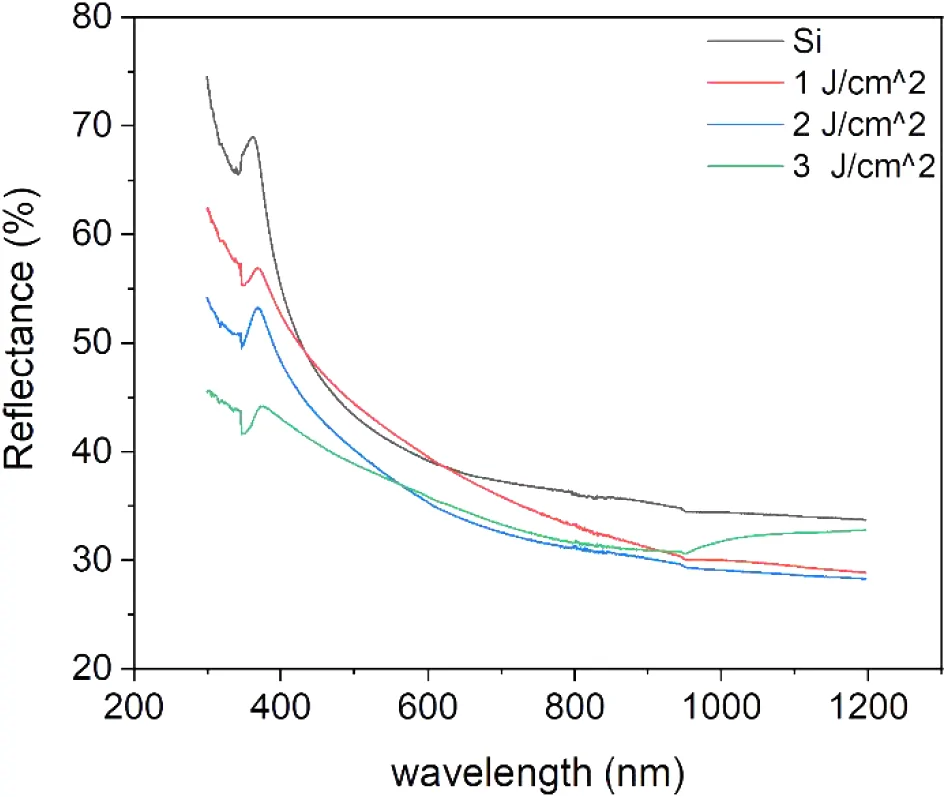
UV–vis reflectance spectra of bare Si and Ge microcone-coated Si substrates fabricated at different laser fluences. Increasing fluence leads to broadband reflectance reduction due to the development of textured Ge microcone arrays.

The UV–Vis reflectance spectra obtained in the present study are consistent with previous reports demonstrating that surface microstructuring effectively suppresses optical reflection in semiconductor materials. For example, Li et al.^33^ fabricated microcone arrays on silicon carbide (SiC) surfaces and achieved infrared reflectance values below 20%, whereas *Chang et al.* ^34^ reported morphology-dependent optical properties in SiGe nanostructures grown on glass substrates, with reflectance decreasing as the surface morphology became more developed. Similarly, the present results reveal a systematic reduction in reflectance with increasing laser fluence, which directly increases the surface density of Ge microcones. The sample deposited at 3 J/cm^2^ exhibited reflectance values of approximately 30–40% over the 550–1200 nm wavelength range, representing a substantial improvement compared with bare Si. Although lower reflectance values have been reported for microstructured SiC and SiGe, those structures generally require multistep fabrication routes involving etching, lithography, or high-temperature processing. In contrast, the present approach produces crystalline Ge microcones directly during PLD in a single deposition step. Furthermore, because the density of the microcones is governed by the deposition conditions and increases with laser fluence, the results suggest that further optimization of the deposition process—such as increasing the number of laser pulses while maintaining the optimal fluence—may further enhance surface coverage and reduce optical reflectance. These findings highlight both the tunability and the scalability of the proposed PLD approach for engineering light-trapping Ge/Si interfaces.

Figure 4 shows the XRD pattern of a representative Ge thin film. All peaks correspond to the Ge diamond-like cubic structure (ICDD: 00-004-0545), indicating the absence of secondary phases or impurities (e.g., GeO_2_). The (111) peak is noticeably more intense, indicating a preferential crystallographic orientation along that direction. However, the presence of the remaining planes at lower intensities could be interpreted as indicative of a buffer layer between the substrate and the microcones, which can be observed in the cross-sectional views in Figure 2a–c.

**Figure 4:**
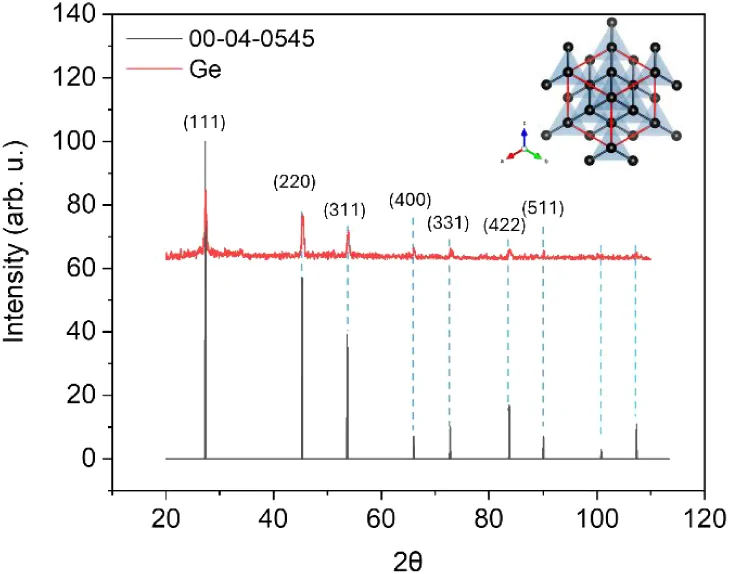
Results of an X-ray diffraction study of a Ge film deposited on Si. Experimental pattern is shown in red, and reference pattern for cubic Ge is shown in black (ICDD 00-04-0545).

## 4 Conclusions

A single-step pulsed laser deposition (PLD) route was demonstrated to fabricate crystalline Ge microcones on Si(001) substrates, with morphology effectively controlled by laser fluence. Increasing the fluence from 1 to 3 J/cm^2^ produced a systematic evolution of the microcone geometry, with cone height increasing from 190 ± 10 nm to 5.8 ± 0.1 μm, base diameter from 360 ± 10 nm to 16.5 ± 0.1 μm, and surface density from (1.07 ± 0.14) × 10^4^ cm^–2^ to (5.31 ± 0.29) × 10^4^ cm^–2^. Based on the available literature, Ge microcones with this distinctive circular-base morphology have not previously been reported using PLD. These morphological changes were directly reflected in the optical response, with the sample deposited at 3 J/cm^2^ exhibiting broadband reflectance of 30–40% over the 550–1200 nm wavelength range, representing a substantial reduction compared with bare Si. The results demonstrate that laser fluence is a simple and effective control parameter for tailoring the morphology, surface coverage, and optical performance of Ge microcones grown by PLD. Furthermore, the proposed droplet-mediated growth mechanism provides a plausible explanation for the observed morphological evolution, while the direct fabrication of crystalline Ge microcones through a catalyst-free, mask-free, single-step deposition process highlights the simplicity and scalability of the approach. These findings establish PLD as a versatile strategy for engineering light-trapping Ge/Si interfaces with tunable optical properties, providing a promising platform for future infrared photonic, optoelectronic, and photovoltaic applications.

## Abbreviations

Ge: germanium
Si: silicon
PLD: Pulsed Laser Deposition
